# Sudden hemothorax as a rare initial manifestation of bronchiectasis under a direct oral anticoagulant

**DOI:** 10.1186/s40792-022-01536-0

**Published:** 2022-09-23

**Authors:** Hikaru Nakayama, Asuka Uebayashi, Shota Yagi, Shuhei Iizuka, Yoshiro Otsuki, Toru Nakamura

**Affiliations:** 1 Department of General Thoracic Surgery, Seirei Hamamatsu General Hospital, 2-12-12, Sumiyoshi, Nakaku, Hamamatsu, Shizuoka 430-8558 Japan; 2 Department of Respiratory Medicine, Seirei Hamamatsu General Hospital, 2-12-12, Sumiyoshi, Nakaku, Hamamatsu, Shizuoka 430-8558 Japan; 3 Department of Pathology, Seirei Hamamatsu General Hospital, 2-12-12, Sumiyoshi, Nakaku, Hamamatsu, Shizuoka 430-8558 Japan

**Keywords:** Bronchiectasis, Hemothorax, Direct oral anticoagulant

## Abstract

**Background:**

A hemothorax as the initial manifestation of bronchiectasis is extremely rare. We report a case of a sudden hemothorax due to exacerbation of clinically latent bronchiectasis under a direct oral anticoagulant.

**Case presentation:**

A 77-year-old woman presented with chest pain and a fever noted since the day before. She had stage G3 chronic kidney disease and received edoxaban for paroxysmal atrial fibrillation. She had no history of trauma or respiratory symptoms. A chest computed tomography revealed a mass with a surrounding opacity in the right lower lobe with a pleural effusion. Conservative treatment was chosen because of the stable vital signs and her respiratory condition. Her oxygen saturation dropped 7 h later with progressive anemia. Repeated chest computed tomography showed a worsening pulmonary opacity and pleural effusion. She underwent a right lower lobectomy successfully. The histopathological findings suggested that the preceding infection of the subpleural focal bronchiectasis caused the bleeding. In addition, a steep caliber change between the subpleural focal bronchiectasis and proximal normal branch may have caused an intraluminal pressure gradient resulting in a peripheral discharge causing a pleural rupture with a hemothorax.

**Conclusion:**

The sudden hemothorax could have been the initial manifestation of bronchiectasis. Particular attention should be paid to peripherally localized bronchiectasis even if it is without any clinical symptoms, especially in patients with a comorbidity such as a susceptibility to infections and the use of direct oral anticoagulants.

## Background

Bronchiectasis often manifests as a persistent productive cough, and hemoptysis as the initial manifestation is less common [1, 2]. Moreover, a hemothorax as the initial manifestation of bronchiectasis is extremely rare. We report a case of a hemothorax due to an exacerbation of clinically latent bronchiectasis under a direct oral anticoagulant (DOAC).

## Case presentation

A 77-year-old woman presented with chest pain and a fever noted since the day before. She had stage G3 chronic kidney disease (CKD) and received edoxaban for paroxysmal atrial fibrillation with a CHADs score of 3. She had no history of trauma or respiratory symptoms.

**Fig. 1:**
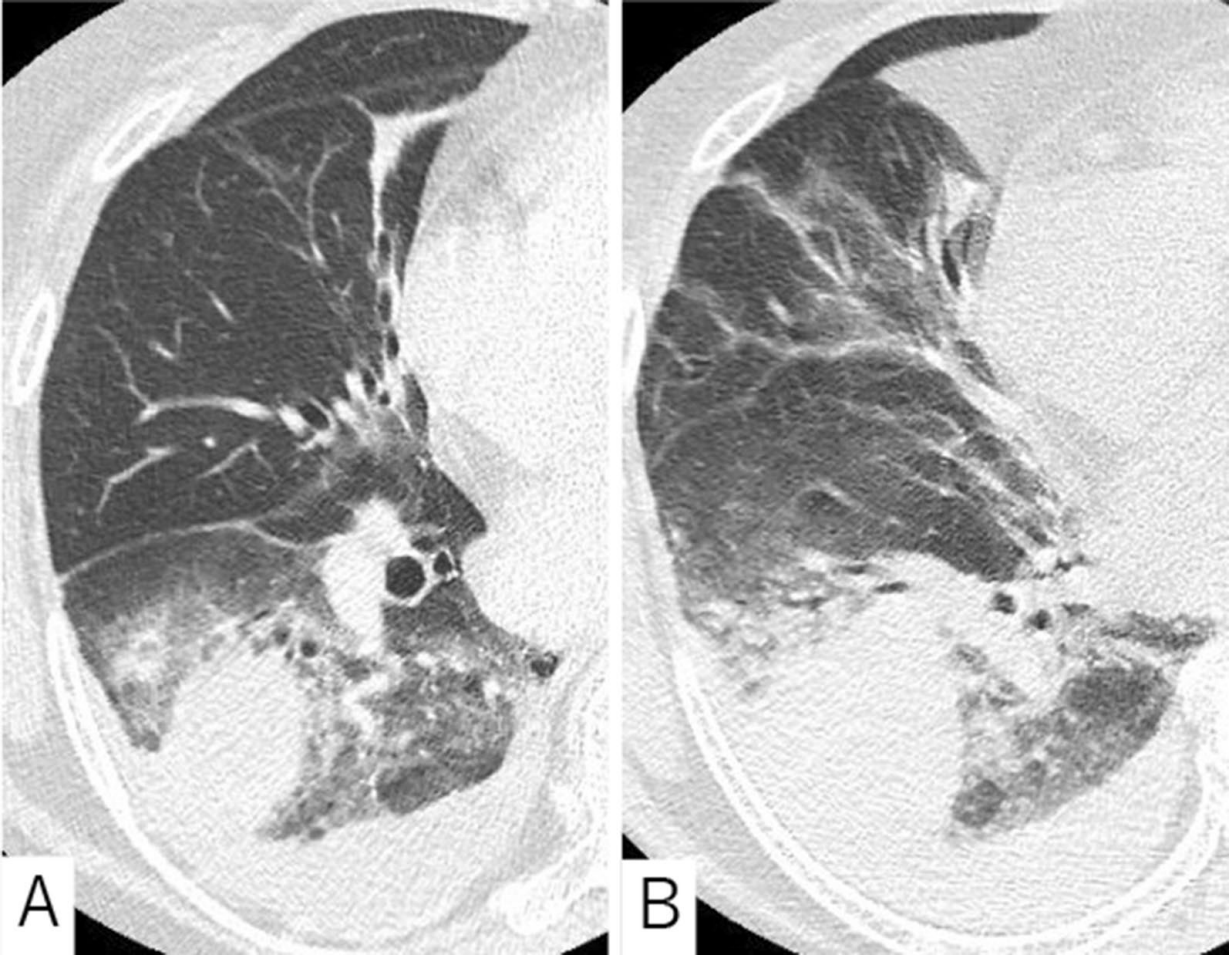
**A** A chest CT on arrival showing a mass with surrounding opacity in the right lower lobe with a pleural effusion. **B** A chest CT 7 h later showing a worsening pulmonary opacity and a pleural effusion

A chest computed tomography (CT) revealed a mass with surrounding opacity in the right lower lobe and a pleural effusion (Fig. 1A). Alveolar bleeding with a hemothorax due to a rupture of a pulmonary artery aneurysm was suspected. Conservative treatment was chosen because of the stable vital signs and her respiratory condition with an oxygen saturation (SpO_2_) of 94% on room air upon arrival.

However, the SpO_2_ dropped to 90% 7 h later with progressive anemia (hemoglobin level from 12.9 to 10.7 mg/dL). A repeated chest CT also showed a worsening pulmonary opacity and a pleural effusion (Fig. 1B).

Interventional radiology was not applicable because of the lack of any active contrast extravasation on the CT, and therefore an emergency surgery was indicated.

**Fig. 2:**
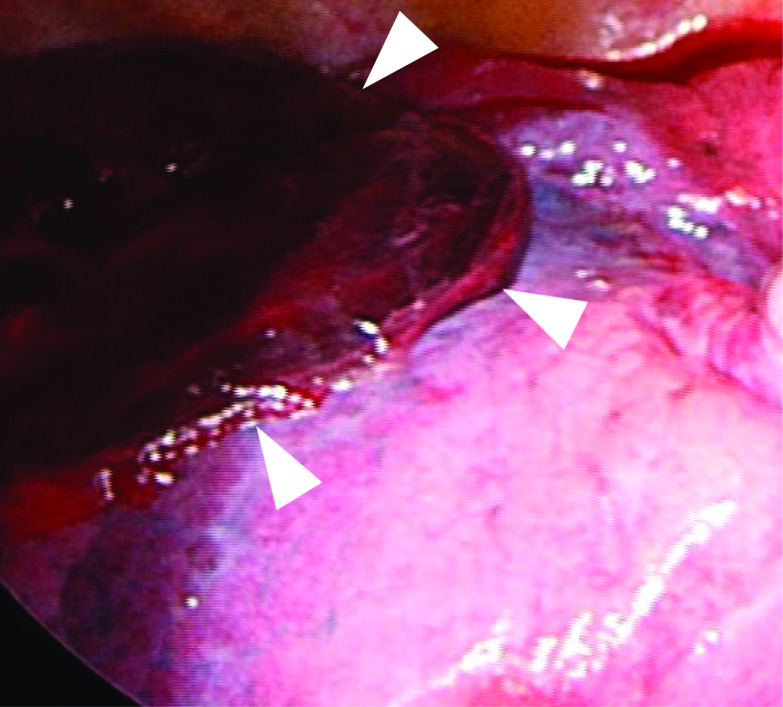
The pleural surface of the lower lobe was found to be torn and covered with a blood clot (arrows) during the surgery

After evacuating the bloody pleural fluid via aright posterolateral thoracotomy, the pleural surface of the lower lobe was found to be torn and overlaid with blood clots (Fig. 2). She underwent a right lower lobectomy successfully with an improvement in her oxygenation. The operation time was 120 min and the total amount of bleeding was 500 g including the pre-existing pleural fluid. The postoperative course was uneventful, and she was discharged on the third postoperative day.

**Fig. 3:**
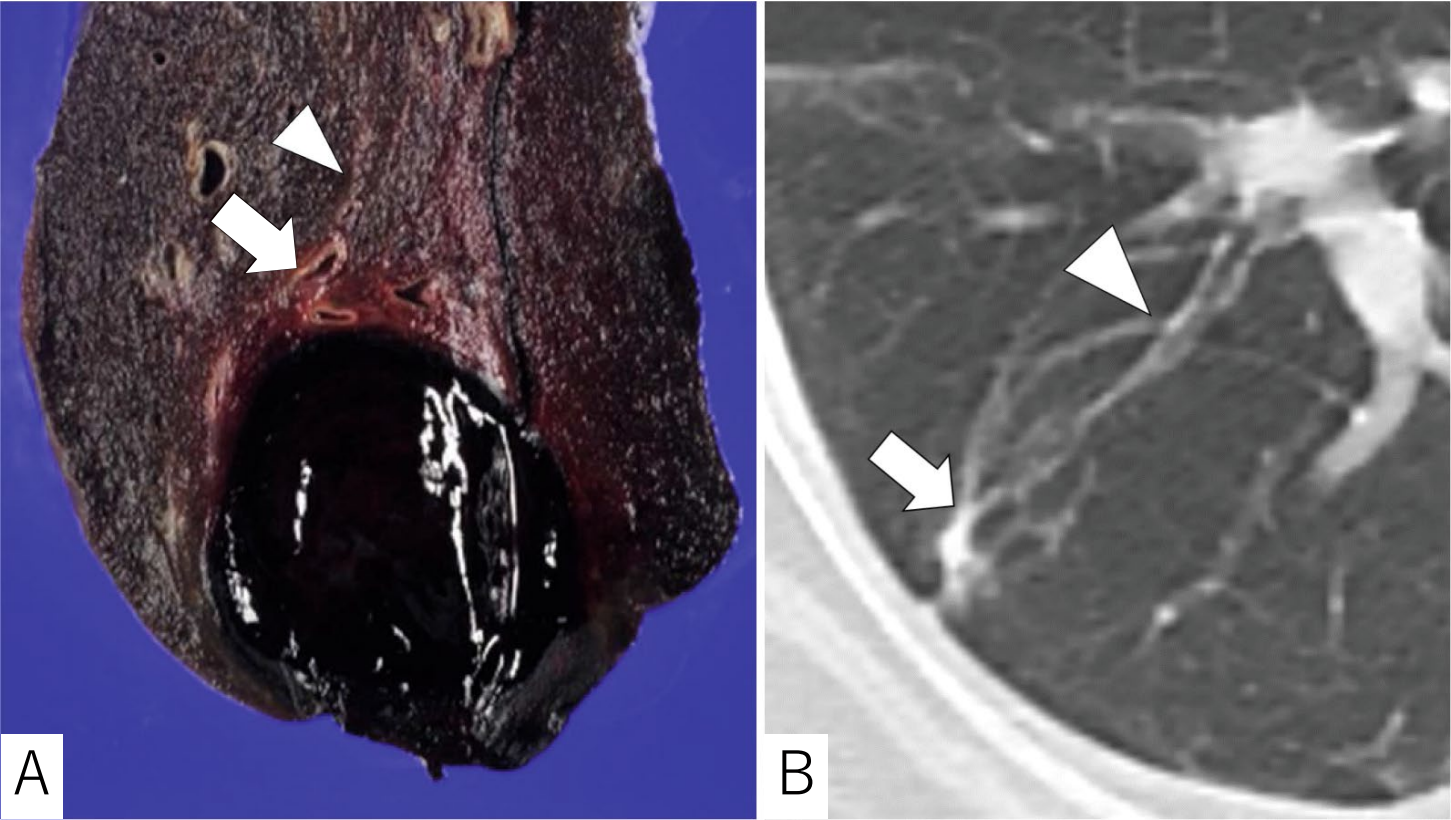
**A** The cut surface of the specimen showing a focally dilated bronchiole (arrow) with a normal caliber proximal branch (arrowhead). The blood clot was deemed to have become dislodged from the dilated bronchiole. **B** A chest CT a year prior revealed a steep caliber change between the dilated (arrow) and normal caliber bronchiole (arrowhead)

**Fig. 4:**
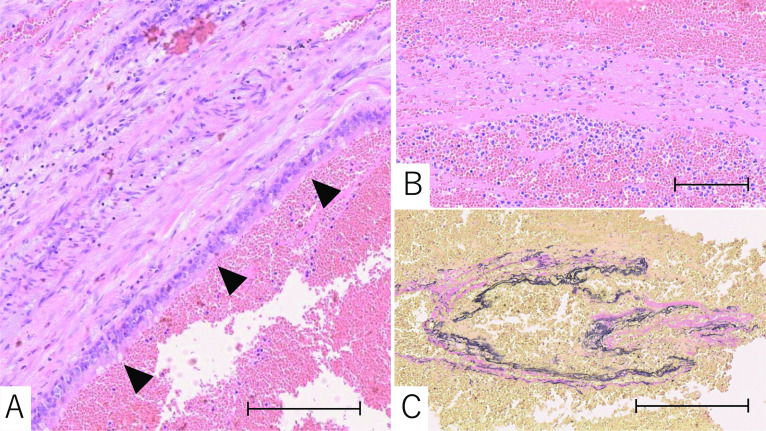
**A** Histopathological findings showing that the primary bleeding site was within the focally dilated bronchiole (scale bar: 200 μm). The arrowheads indicate ciliated bronchial epithelial cells. **B** The dilated bronchiole wall is accompanied by neutrophil infiltration, suggesting a preceding infection (scale bar: 100 μm). **C** Elastica van Gieson staining revealed ruptured bronchial artery in the surrounding bronchiole wall (scale bar: 200 μm)

The cut surface of the specimen revealed a subpleural expansive blood clot from the focally dilated bronchiole distal to the normal bore branch (Fig. 3A). A chest CT a year prior had revealed a steep caliber change in the bronchiole between the subpleural focal bronchiectasis and proximal normal branch (Fig. 3B). The histopathological findings showed that the focally dilated bronchiole wall was accompanied by neutrophil infiltration, suggesting that the preceding infection had developed and involved the surrounding bronchial arteries and caused the bleeding (Fig. 4).

## Discussion

The most frequent initial manifestation of bronchiectasis is a persistent productive cough and hemoptysis is less common. Furthermore, a hemothorax caused by bronchiectasis is extremely rare. The present case developed an unexpected sudden hemothorax as the initial manifestation of bronchiectasis requiring surgery.

The histopathological findings suggested that the preceding infection of the focal bronchiectasis caused the bleeding. Despite the absence of any respiratory symptoms before the onset, she had been at risk both for an infection and bleeding because of stage G3 CKD [3, 4] and DOAC administration [5–10]. That medical background may have accelerated the bleeding.

In addition, the steep caliber change between the bronchiectasis and proximal normal bore bronchiole was deemed the cause of the hemothorax, not the hemoptysis. Sudden bleeding at the distal aspect of this bronchiole caliber gap may have caused an intraluminal pressure gradient resulting in a peripheral discharge causing the pleural rupture with a hemothorax. A chest CT a year prior and the gross findings of the surgical specimen supported this hypothesis. The predominantly intraluminal bleeding also obstructed the proximal bronchiole and prevented the air from leaking, resulting in hemothorax without a pneumothorax. A combined medical condition and anatomical anomaly adversely affected the rare initial manifestation of bronchiectasis in the present case. A lobectomy was deemed essential for hemostasis but a sublobar resection might also have been applicable during an earlier stage of the disease.

## Conclusion

The sudden hemothorax could have been the initial manifestation of bronchiectasis even in asymptomatic patients with a complex comorbidity and bronchiole anomaly. Particular attention should be paid to peripherally localized bronchiectasis even if it is without any clinical symptoms, especially in patients with a comorbidity such as a susceptibility to infections and the use of DOACs.

## Data Availability

Not applicable.

## Abbreviations

CT: Computed tomography
DOAC: Direct oral anticoagulants
CKD: Chronic kidney disease
